# PAQR4 promotes chemoresistance in non-small cell lung cancer through inhibiting Nrf2 protein degradation

**DOI:** 10.7150/thno.43142

**Published:** 2020-02-19

**Authors:** Peifang Xu, Liping Jiang, Yang Yang, Mengge Wu, Baiyang Liu, Yulin Shi, Qiushuo Shen, Xiulin Jiang, Yaomei He, Dating Cheng, Qiuxia Xiong, Zuozhang Yang, Lincan Duan, Jie Lin, Song Zhao, Peng Shi, Cuiping Yang, Yongbin Chen

**Affiliations:** 1 School of Life Sciences, University of Science and Technology of China, Hefei, Anhui 230027, China.; 2 Key Laboratory of Animal Models and Human Disease Mechanisms of Chinese Academy of Sciences & Yunnan Province, Kunming Institute of Zoology, Kunming, Yunnan 650223, China.; 3 Kunming College of Life Science, University of Chinese Academy of Sciences, Beijing, 100049, China.; 4 Department of Thoracic Surgery, the First Affiliated Hospital of Zhengzhou University, Zhengzhou 450052, China.; 5 Kunming Medical University, Kunming 650223, China.; 6 State Key Laboratory of Genetic Resources and Evolution, Kunming Institute of Zoology, Chinese Academy of Sciences, Kunming, Yunnan 650223, China.; 7 Center for Excellence in Animal Evolution and Genetics, Chinese Academy of Sciences, Kunming, Yunnan 650223, China.

**Keywords:** Progestin and AdipoQ Receptor 4, non-small cell lung cancer, Nrf2, Keap1, ubiquitination

## Abstract

**Purpose:** Lung cancer is the leading cause of cancer related deaths worldwide. We have previously identified many differentially expressed genes (DEGs) from large scale pan-cancer dataset using the Cross-Value Association Analysis (CVAA) method. Here we focus on Progestin and AdipoQ Receptor 4 (PAQR4), a member of the progestin and adipoQ receptor (PAQR) family localized in the Golgi apparatus, to determine their clinical role and mechanism in the development of non-small cell lung cancer (NSCLC).

**Methods:** The protein expression profile of PAQR4 was examined by IHC using tissue microarrays, and the effects of PAQR4 on cell proliferation, colony formation and xenograft tumor formation were tested in NSCLC cells. Real-time RT-PCR, co-immunoprecipitation (co-IP) and GST-pulldown assays were used to explore the mechanism of action of PAQR4.

**Results:** We provided evidence showing that PAQR4 is increased in NSCLC cancer cell lines (A549, H1299, H1650, H1975, H358, GLC-82 and SPC-A1), and identified many mutations in PAQR4 in non-small cell lung cancer (NSCLC) tissues. We demonstrated that PAQR4 high expression correlates with a worse clinical outcome, and that its knockdown suppresses cell proliferation by inducing apoptosis. Importantly, overexpressed PAQR4 physically interacts with Nrf2 in NSCLC cells, blocking the interaction between Nrf2 and Keap1.

**Conclusion:** Our results suggest that PAQR4 depletion enhances the sensitivity of cancerous cell to chemotherapy both *in vitro* and xenograft tumor formation *in vivo*, by promoting Nrf2 protein degradation through a Keap1-mediated ubiquitination process.

## Introduction

Lung cancer is the predominant cause of cancer related deaths 1. Within the main types of lung cancers, small cell lung cancer (SCLC) and non-small cell lung cancer (NSCLC), NSCLC accounts for approximately 85% of all lung cancer, with a poor 5-year survival of only ~15%. NSCLC includes lung adenocarcinoma (ADC), lung squamous cell carcinoma (SCC) and large-cell lung carcinoma 1. Despite the huge advances in treatment options including surgery, chemotherapy, radiation and targeted therapies, prognosis remains poor because of the presence of locally advanced metastatic tumors in most patients at the time of diagnosis 2. NSCLC patients are treated uniformly with a one-size-fits-all approach - Platinum-based chemotherapy, where cisplatin or carboplatin is used in combination with gemcitabine, vinorelbine, pemetrexed or taxanes (docetaxel or paclitaxel) 3. However, patients may also benefit from other therapeutic approaches that involve interruptions of essential signaling pathways important for NSCLC development and progression. Clinical trials with tyrosine kinase inhibitors (TKIs) targeted to mutant EGFR or ALK-fusion proteins, show a significant increase of the 5-year survival rate of NSCLC. Importantly, most patients with NSCLC are sensitive to chemotherapy at the early stage, but show drug resistance in the late stage, and the occurrence of inevitable drug resistance requires the identification of novel targets and the development of personalized medicine in future 4, 5.

After decades of research, various cancer cell intrinsic drug resistance mechanisms have been identified, e.g., activation of multidrug-resistance (MDR)-associated protein transporters, resistance to apoptosis or senescence signaling pathways and altered expression of detoxifying enzymes 6-9. In particular, the overexpression of glutathione S-transferases (GSTs) may reduce the reactivity of various anticancer drugs 10. The increase of GST levels is regulated by transcription factor Nrf2 (nuclear factor erythroid 2-related factor 2). Nrf2 is a master transcriptional activator that drives the cellular response to combat a variety of stresses including oxidative stress, proteotoxic stress and electrophilic insults 11-14. Under basal conditions, Nrf2 is constantly ubiquitinated by Keap1 (Kelch-like ECH-associated protein 1) and degraded through the proteasome pathway 11. Exposure of cells to oxidative stress and electrophilic insult inactivates Keap1 and stabilizes Nrf2. Nrf2 then translocates into the nucleus and binds to the ARE (antioxidant response element), activating the transcription of many cytoprotective genes that encode detoxifying enzymes and antioxidant proteins 13. The induction of these genes confers resistance against xenobiotic and oxidative stresses. Recently, the constitutive stabilization of Nrf2 was found in various human cancers 15-19, and cancers with high Nrf2 levels are associated with poor prognosis 17, 18. Consistently, the role of Nrf2 in determining the efficacy of cisplatin was also demonstrated in ovarian cancer 20. Moreover, many Keap1 mutations or loss of heterozygosity in the Keap1 locus has been identified in lung cancer 6. Therefore, identification of new factors involved in Keap1-Nrf2 signaling pathways may benefit NSCLC patients with drug resistance in future.

We recently used a new method, Cross-Value Association Analysis (CVAA), to analyze the pan-cancer dataset, and identified numerous new differentially expressed genes (DEGs) 21, including Progestin and AdipoQ Receptor 4 (PAQR4), a member of the progestin and adipoQ receptor (PAQR) family, which includes 11 members (PAQR1 to PAQR11) in the human genome 22. The conservation of the PAQR proteins between human and mouse orthologues is high 22. The prototypes in this family are PAQR5-8 that function as membrane receptors for progesterone 23, as well as AdipoR1 and AdipoR2 (i.e., PAQR1 and PAQR2) are surface receptors for adiponectin and play important roles in metabolic regulation 24. Especially, PAQR3, the closest homologue of PAQR4, has been recently discovered as a novel tumor suppressor deregulated in various types of human cancers including colon cancer, gastric cancer, bladder cancer, liver cancer, osteosarcoma, breast cancer, and laryngeal squamous cell carcinoma 25-30. PAQR3 functions as a tumor suppressor mainly due to its negative regulation of the Ras/Raf/MEK/ERK signaling cascade 25. Moreover, PAQR3 was identified as an adaptor protein that facilitates Keap1-Nrf2 complex formation leading to Nrf2 degradation and modulates antioxidant response 31. Recent studies demonstrated that CDK4 protein level is controlled by the antagonistic actions between PAQR4 and SKP2, which contribute to the regulation of cell proliferation and tumorigenesis 32-34. However, whether PAQR4 is involved in antioxidant or drug resistance functions in cancers remains elusive. In this study, we explored the potential antioxidant function of PAQR4 in NSCLC.

## Results

### PAQR4 is highly expressed in NSCLC

**Figure 1:**
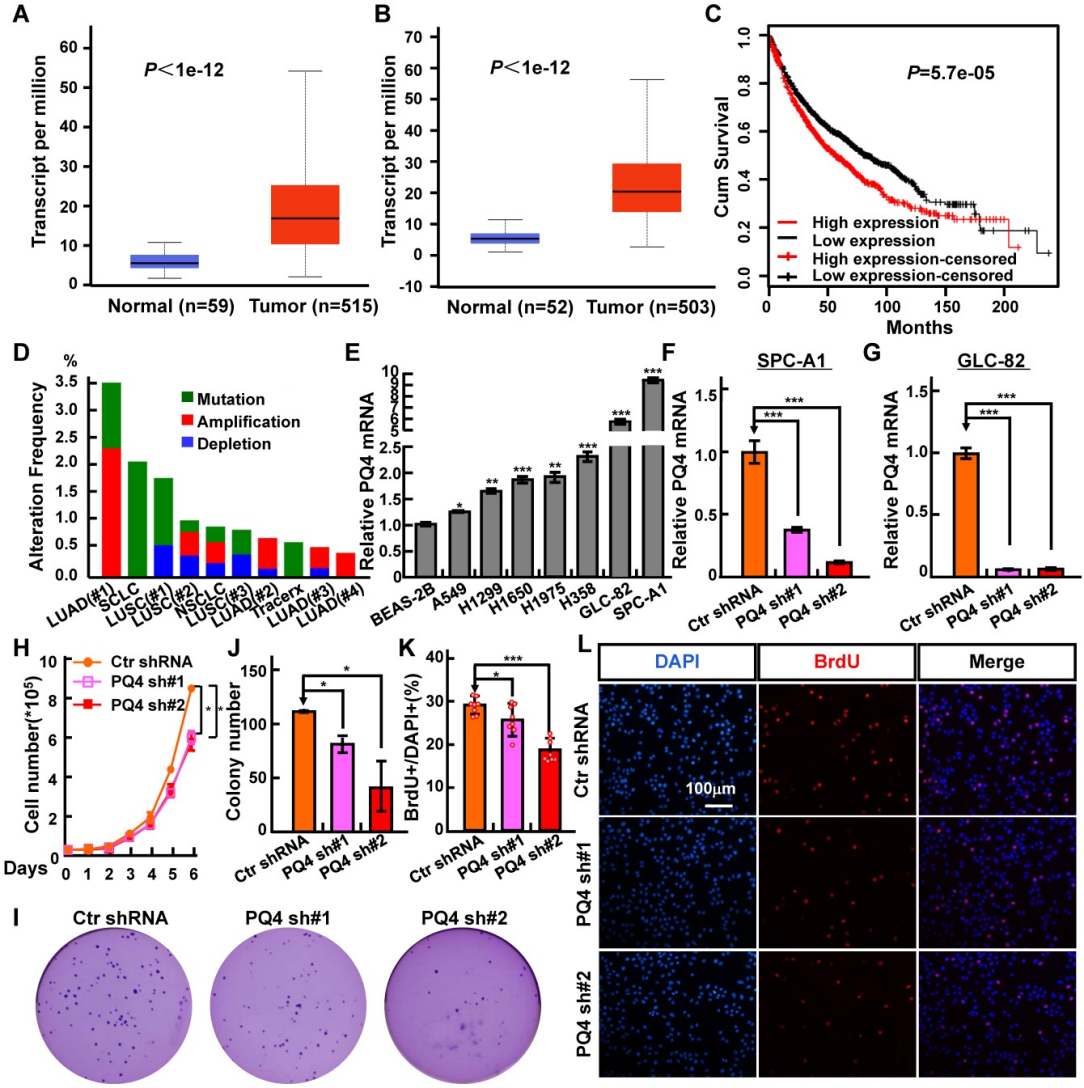
** PAQR4 is highly expressed in NSCLC. (A-B)** PAQR4 is highly expressed in lung adenocarcinoma-LUAD (A) and lung squamous cell carcinoma-LUSC (B). **(C)** PAQR4 high expression in NSCLC leads a significantly worse survival rate than its low expression patients.** (D)** PAQR4 is frequently mutated in NSCLC. Mutation: green; deletion: blue; amplification: red. **(E)** PAQR4 mRNA was increased in various NSCLC cell lines: A549, H1299, H1650, H1975, H358, GLC-82 and SPC-A1, compared with control cell line BEAS-2B. **(F-G)** Establishment of PAQR4 knockdown in SPC-A1 (F) and GLC-82 (G) cell lines and verified by real-time RT-PCR. **(H-L)** PAQR4 knockdown dramatically inhibits SPC-A1 cell proliferation (H), colony formation (I, J) and BrdU incorporation abilities (K, L). Means±SEM, * *P* <0.05; ** *P* <0.01; *** *P* <0.001; *t*-test. PQ4=PAQR4. sh#1=shRNA#1, sh#2=shRNA#2.

In our previous study, we developed a new method called CVAA (Cross-Value Association Analysis), which functions without a normalization and distribution assumption. We applied it to large-scale pan-cancer transcriptome data generated by The Cancer Genome Atlas (TCGA) project, and successfully discovered numerous new differentially expressed genes (DEGs) 21. PAQR4 is one of these DEGs. It is a member of the PAQR family involved in the regulation of a number of biological processes including metabolism and cancer development 23-30. To investigate the potential roles of PAQR4 in tumorigenesis, we examined its expression in various cancer types using gene expression profiling interactive analysis 35, and found that PAQR4 is highly expressed in many cancer types **(Figure S1A; Table 1)**. In addition, we surveyed PAQR4 protein expression in NSCLC (including lung SCC and ADC) and noncancerous control lung tissues (NCLT) by immunohistochemical staining (IHC) using a tissue microarray (**Figure S1B-C; Table 2**). The percentage of positive PAQR4 expression was significantly higher in ADC tissues (65.9%) than that in NCLT tissues (40.8%). Furthermore, we found that PAQR4 is upregulated in both lung adenocarcinoma and lung squamous cell carcinoma by UALCAN software **(Figure 1A-B)** 36.

Next, we found that PAQR4 high expression correlates with a worse overall survival rate, using a Kaplan Meier plotter containing the affymetrix gene expression dataset for lung cancer **(Figure 1C)** 37. Finally, to examine whether PAQR4 is also highly expressed in NSCLC cancerous cells, we analyzed its relative mRNA expression in normal human bronchial epithelium cell line (BEAS-2B) and NSCLC cancerous cell lines (A549, H1299, H1650, H1975, H358, GLC-82 and SPC-A1). Consistent with our findings in the database **(Figure S1A)**, we found that PAQR4 is up-regulated in all NSCLC cancerous cell lines, with relatively higher expression levels in SPC-A1 and GLC-82 **(Figure 1E)**. Due to the higher expression of PAQR4 in NSCLC cancerous cell lines we hypothesized that PAQR4 might also be frequently mutated in NSCLC. Indeed, we identified many PAQR4 mutants in NSCLC by applying the cBioPortal web resource 38 **(Figure 1D; Table 3-4)**, suggesting that PAQR4 plays potential important role in lung homeostasis.

### PAQR4 knockdown inhibits NSCLC cell proliferation and induces cellular apoptosis

NSCLC cell lines SPC-A1 and GLC-82 were chosen to further validate the functional roles of PAQR4. We inhibited PAQR4 mRNA expression with two lenti-viral shRNAs **(Materials and Methods)**, and verified the inhibition efficiency by real-time RT-PCR **(Figure 1F-G)**. As expected, stable knockdown of PAQR4 inhibited SPC-A1 and GLC-82 cell proliferation and colony formation *in vitro*, and this effect could be rescued by PAQR4 overexpression **(Figure 1H-J and Figure S1D, E, I, J)**. Indeed, we observed that PAQR4 knockdown significantly decreased the ratio of BrdU-positive cells in SPC-A1 and GLC-82 **(Figure 1K-L and Figure S1F-G)**. Previous findings indicated that PAQR4 promotes cell proliferation by stabilizing CDK4 33, 34. However, we analyzed the cell cycle transition by flow cytometry, and did not find significant cell cycle modulation after PAQR4 knockdown, or change in CDK2 and CDK4 protein stabilities **(Figure S1H, K)**, suggesting a cellular context dependent role for PAQR4.

**Figure 2:**
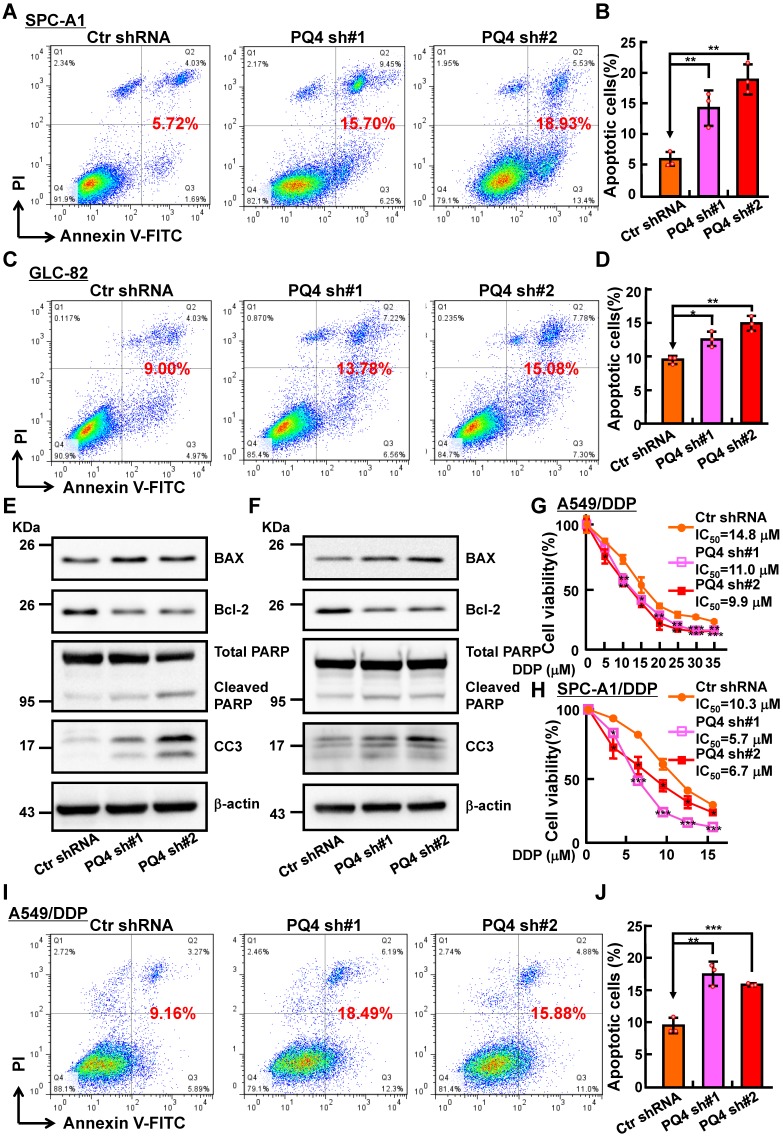
** PAQR4 knockdown promotes cisplatin (DDP) induce cellular apoptosis**. **(A-D)** PAQR4 knockdown promotes cisplatin (DDP) induced cellular apoptosis in SPC-A1 (A-B) and GLC-82 (C-D) cells. After 24 h treatment with 8 μM DDP, indicated cells were stained with Annexin V/PI, and the percentage of apoptotic cells was assessed by flow cytometry (A, C). (B) Quantification data for (A). (D) Quantification data for (C).** (E-F)** Effects of DDP on cellular apoptosis of indicated SPC-A1 (E) and GLC-82 cell lines (F) examined by western blot. 48 h after DDP treatment, indicated total extracts were probed with indicated antibodies: PARP, CC3, Bcl-2, BAX and β-actin.** (G-H)** PAQR4 knockdown promotes DDP induced cellular apoptosis in A549/DDP cells (G) and SPC-A1/DDP cells (H) detected by SRB assay. **(I-J)** PAQR4 knockdown promotes DDP induced cellular apoptosis in A549/DDP cells. After 24 h treatment by 20 μM DDP, indicated cells were stained with Annexin V/PI, and the percentage of apoptotic cells were assessed by flow cytometry (I). (J) Quantification data for (I). Means±SEM, * *P* <0.05; ** *P* <0.01; *** *P* <0.001; *t*-test.

To decipher how PAQR4 regulates cell proliferation without affecting cell cycle transition, we decided to examine the potential role of PAQR4 on regulating cellular apoptosis. Indeed, increased cellular apoptosis in PAQR4 knockdown cells was detected by comparison with that in scramble shRNA control cells **(Figure S2A-C)**. Consistently, increased cisplatin (DDP), Carboplatin (CBP) or Etoposide (ETO) induced cellular apoptosis was observed in PAQR4 knockdown cells, and confirmed by Cleaved Caspase 3 (CC3) and PARP western blot **(Figure 2A-F and Figure S2D-F)**. *In vitro*, we also found that PAQR4 is upregulated in cisplatin-resistant A549 (A549/DDP) and SPC-A1 (SPC-A1/DDP) cell lines, whereas knockdown of PAQR4 in both cells inhibited cellular viability **(Figure 2G-J and Figure S2G-J)**. The above data suggest that PAQR4 could be used as a novel therapeutic target in combination with platinum-based chemotherapy in the future.

### Inhibition of xenograft tumor formation *in vivo*

**Figure 3:**
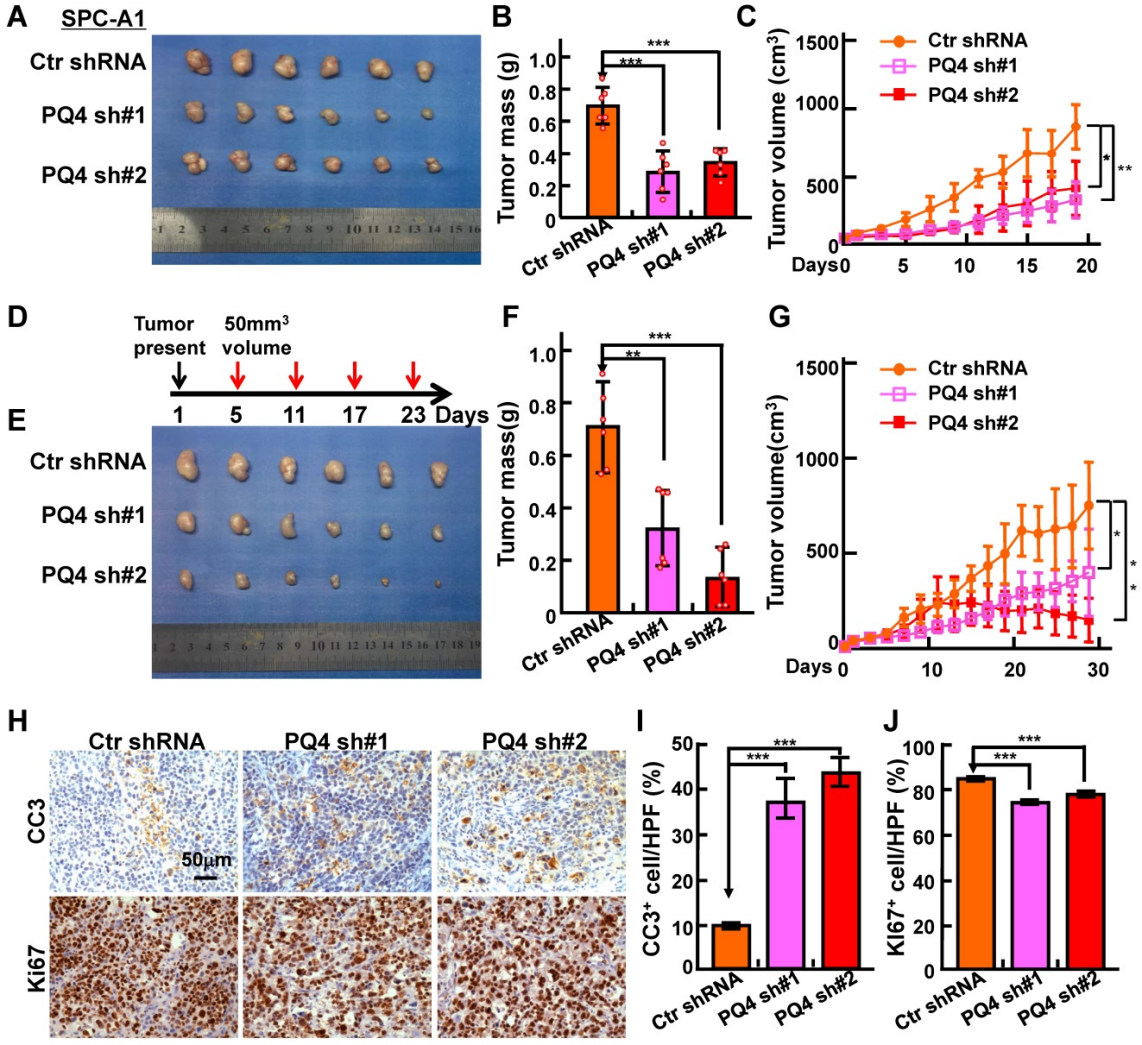
** PAQR4 knockdown inhibits xenograft tumor formation and increases cisplatin sensitivity *in vivo*. (A-C)** PAQR4 knockdown inhibits xenograft tumor formation *in vivo*. Representative xenograft tumor images (A), tumor masses (B) and tumor volumes (C) are shown for indicated groups. SPC-A1 cell lines were used. Tumor masses were collected and photographed at the end of the experiment, tumor weights were weighted using electronic scales and tumor volumes measured with a sliding caliper every two days using the formula (L×W^2^)/2. **(D-G)** PAQR4 knockdown increases xenograft tumor sensitivity to cisplatin. Schematic diagram shows the experimental strategy for DDP (7 mg/kg) treatment in nude mice (D). In detail, after xenograft tumors reached about 50 mm^3^ in size, mice were injected with DDP by intraperitoneal injection every 6 days. At the end of the experiment, representative xenograft tumor images (E), tumor masses (F) and tumor volumes (G) were recorded for the indicated groups. SPC-A1 cell lines were used. **(H-J)** Representative IHC staining of Ki67 and Cleaved Caspase 3 (CC3) for indicated xenograft tumors after DDP treatment (H). Scale bar: 50 μm. The quantification data for CC3 (I) and Ki67 (J) are included. The percentages of positive cells were counted using Image pro plus 6.0, the percentage of positive cells in every image was calculated using the formula: number of positive cells/total number of cells. Means±SEM, * *P* <0.05; ** *P* <0.01; *** *P* <0.001; *t*-test.

To test the potential roles of PAQR4 *in vivo*, we performed a xenograft tumor formation assay using nude mice. Male nude mice aged 5 weeks were randomly divided into 3 groups, injected with 2 independent PAQR4 stable knockdown cell lines or scramble control shRNA cells (1×10^6^ cells/ subcutaneously), followed by daily monitoring and weighing every other day. As expected, tumor formation, tumor weights and volumes in PAQR4 knockdown groups were dramatically decreased over the control group **(Figure 3A-C and Figure S3A-D)**. To validate the potential drug resistance role of PAQR4 *in vivo*, mice were injected with DDP (7 mg/kg) by intraperitoneal injection every 6 days, after xenograft tumors reached about 50 mm^3^ in size. In line with the findings *in vitro*, dramatically lower proliferation as measured by Ki67 IHC, and higher overall apoptosis indicated by CC3 IHC, in PAQR4 shRNA tumors were detected **(Figure 3D-J)**.

### PAQR4 inhibits Nrf2 degradation by physically interrupting Keap1-Nrf2 complex formation

**Figure 4:**
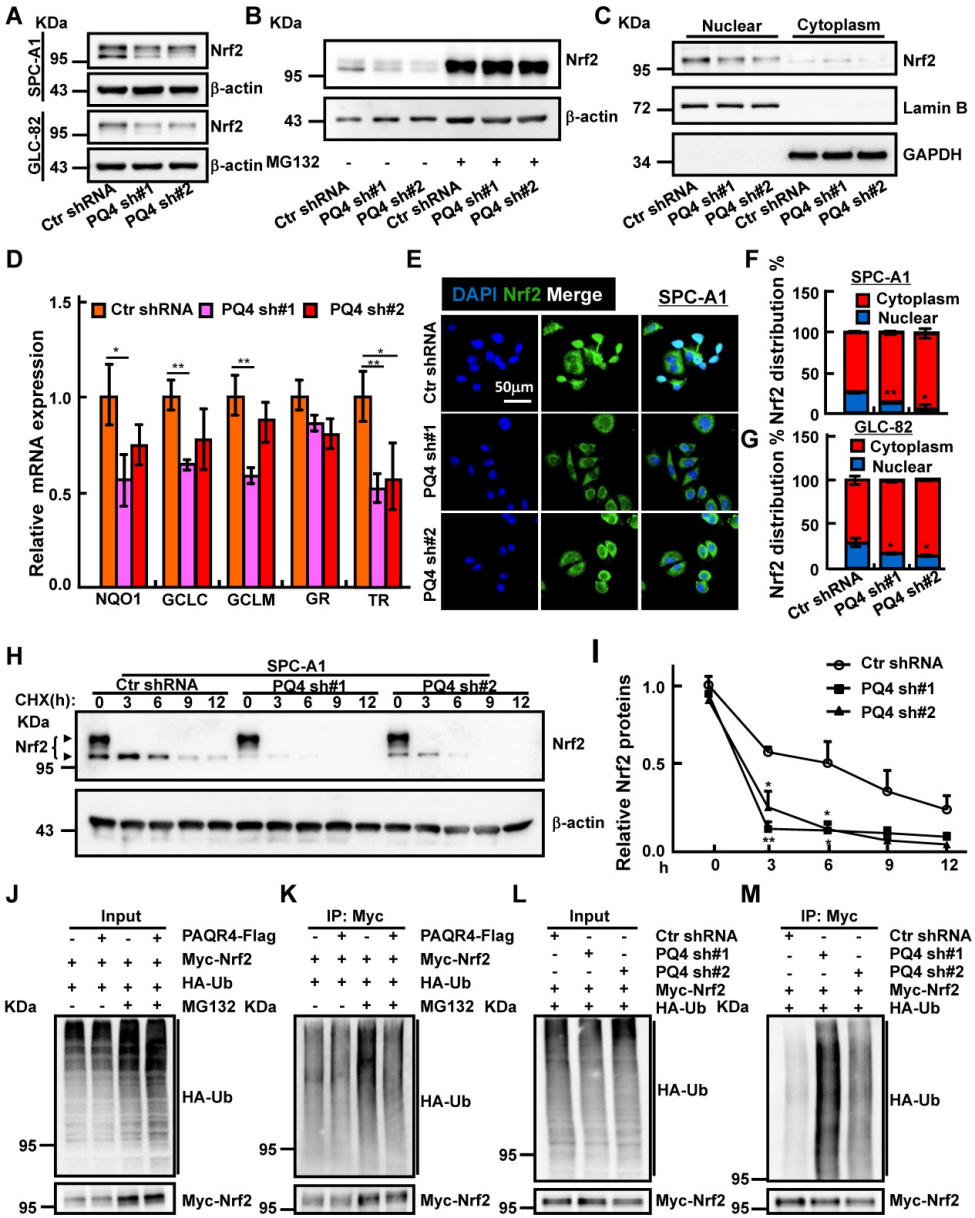
** PAQR4 promotes Nrf2 protein nuclear localization through inhibiting Nrf2 degradation. (A-B)** Total Nrf2 proteins were reduced by PAQR4 knockdown in indicated cells (A), which can be reversed by MG132 -proteasome inhibitor (B). Indicated total extracts were probed with Nrf2 and β-actin antibodies. **(C)** PAQR4 knockdown inhibits nuclear accumulation of Nrf2 proteins in SPC-A1 cells by western blot. Lamin B: nuclear fraction; GAPDH: cytosol fraction. **(D)** Relative mRNA expression of Nrf2 downstream targeted genes after PAQR4 knockdown by real-time RT-PCR in SPC-A1 cells. **(E-G)** Representative images of immunofluorescent staining of Nrf2 protein in indicated cell lines (E). (F) is the quantification data for (E) in SPC-A1 cells, (G) Quantification data for immunostaining of Nrf2 in GLC-82 cells. **(H-I)** Lysates of indicated cell lines treated by cycloheximide (CHX: 100 μg/mL) were examined by western blot with indicated antibodies. (I) Quantification data for (H). The dark arrow heads point to Nrf2 proteins based on previous findings 45. **(J-M)** Analysis of Nrf2 ubiquitination upon PAQR4 overexpression or knockdown was examined in indicated cells by western blot with differential antibodies: HA, Myc. Means±SEM, * *P* <0.05; ** *P* <0.01; *** *P* <0.001; *t*-test.

Antioxidant defense systems regulated by transcription factor Nrf2 has been documented to play an important role in drug resistance mechanisms 39-41. Based on the established role of PAQR3 in facilitating Keap1-Nrf2 complex formation leading to Nrf2 degradation 31, we hypothesized that PAQR4 might also regulate the Nrf2 protein degradation process. Contrary to PAQR4 high expression in NSCLC, PAQR3 expression was decreased in NSCLC **(Figure S4A)** 42. As expected, Nrf2 protein levels were strongly reduced in PAQR4 knockdown cells, which could be reversed by proteasome inhibitor MG132 treatment, whereas the mRNA transcripts of Nrf2 were not regulated by PAQR4 **(Figure 4A-B and Figure S4B, D, E)**, suggesting that PAQR4 inhibits Nrf2 expression at posttranslational level in a ubiquitination-proteasome dependent manner. Consistently, the mRNA expressions of Nrf2 downstream target genes including NADP(H):quinone oxidoreductase 1 (NQO1), glutamate-cysteine ligase catalytic subunit (GCLC), glutamate-cysteine ligase modifier subunit (GCLM), glutathione reductase (GR), thioredoxin reductase (TR were also decreased upon PAQR4 inhibition **(Figure 4D and Figure S4F)** 43. The levels of the nuclear fraction of Nrf2 protein were also significantly reduced by both western blot and immunofluorescence **(Figure 4C, E, F, G and Figure S4C, G)**. In addition, the increased cellular apoptosis in PAQR4 knockdown cells could be rescued by Nrf2 overexpression** (Figure S4H-J).** Furthermore, we investigated the Nrf2 protein degradation rate, and found that depletion of PAQR4 accelerated Nrf2 degradation in SPC-A1 and GLC-82 cells **(Figure 4H-I and Figure S4K-L)**. Furthermore, PAQR4 overexpression abrogates, whereas its depletion promotes, Nrf2 ubiquitination **(Figure 4J-M)**.

**Figure 5:**
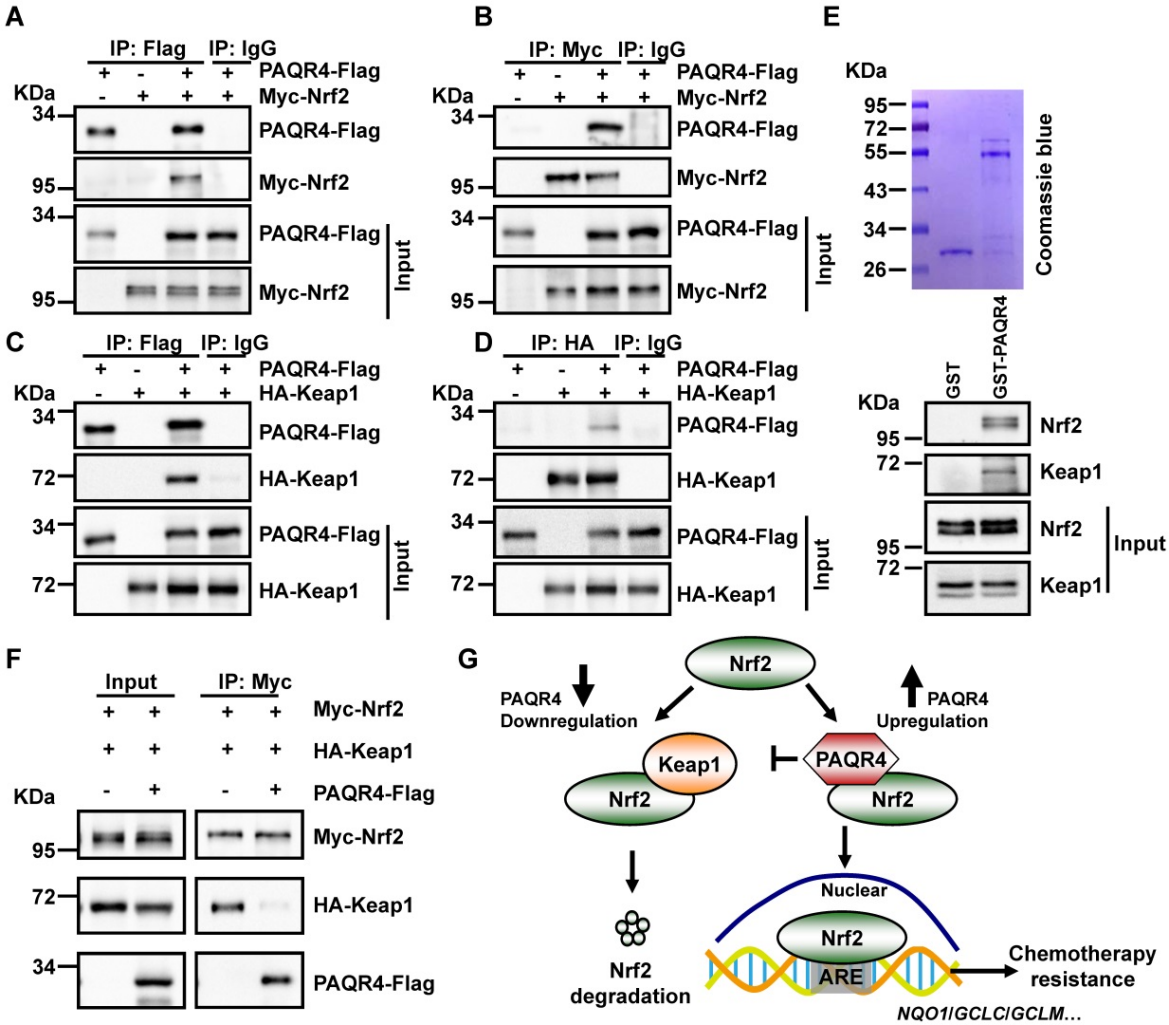
** PAQR4 prevents Nrf2 from ubiquitination by Keap1. (A-B)** PAQR4 interacts with Nrf2. HEK-293T cells were transiently transfected with plasmids as indicated. The cell lysates were used for immunoprecipitation (IP) and immunoblotting (IB) using the antibodies as indicated. **(C-D)** PAQR4 interacts with Keap1. HEK-293T cells were transfected with indicated plasmids and the cell lysates were used for IP and IB.** (E)** PAQR4 interacts with Nrf2 and Keap1 detected by GST pulldown. Bacterially expressed GST-PAQR4 proteins were purified and detected by Coomassie blue staining. SPC-A1 cell lysates were incubated with GST or GST-PAQR4, and pulldown samples were examined by western blot with indicated antibodies. **(F)** PAQR4 decreases the interaction of Nrf2 with Keap1. HEK-293T were transfected with indicated plasmids and the cell lysates were used for IP and IB. **(G)** A model deciphering how PAQR4 promotes chemotherapy resistance mediated by Nrf2. PAQR4 high expression physically competes with Keap1 binding to Nrf2, enhances stabilization and nuclear translocation of Nrf2 proteins, thereby preventing Keap1 mediated ubiquitination and degradation of Nrf2 and resulting in expressions of antioxidant genes and chemotherapy resistance. Means±SEM, * *P* <0.05; ** *P* <0.01; *** *P* <0.001; *t*-test.

Unlike PAQR3, which is specifically localized in the Golgi apparatus 31, PAQR4 mostly colocalizes with Nrf2 or Keap1 in various cell compartments, and only partially in the Golgi **(Figure S5A-C)**. Since the ubiquitination of Nrf2 is regulated by PAQR4, we speculated that PAQR4 might affect the interaction between Nrf2 and Keap1, the E3 ubiquitin ligase of Nrf2 41. Thus, we performed co-immunoprecipitation (co-IP) with cell lysates from Myc-Nrf2 and HA-Keap1 transiently overexpressed HEK-293T, and recapitulated the reciprocal interaction between Nrf2 and Keap1 as documented before **(Figure S5D)**. In addition, the co-IP assay between PAQR4-Flag and Myc-Nrf2/HA-Keap1 validated these interactions **(Figure 5A-D)**. Consistently, we generated and purified the GST-PAQR4 fusion proteins and performed GST pulldown assays, to confirm that GST-PAQR4 binds to the endogenous Nrf2 and Keap1 proteins **(Figure 5E)**. Importantly, the physical interaction between Nrf2 and Keap1 was reduced when PAQR4 was co-expressed in HEK-293T **(Figure 5F)**, suggesting that PAQR4 antagonizes with Keap1 to keep Nrf2 from protein degradation using the ubiquitination-proteasome signaling pathway.

## Discussion

Over the last decade, the genetic backgrounds of cancers have been determined and tumors treated in a tailor made fashion. Remarkable advances in the development of predictive biomarkers and specific targeted small molecule inhibitors have led to improvements in some NSCLC patients' survival rates and quality of life 44. Unfortunately, there is still no effective biomarker for the majority of NSCLC patients, thus chemotherapy is their first choice treatment option. However, the problem of recurrent drug or chemotherapy resistance remains as a major challenge.

In this study **(Figure 5G)**, we identified that PAQR4, a member of the progestin and adipoQ receptor (PAQR) family, and also the closest homologue of PAQR3 25-30, is highly expressed in NSCLC cancerous tissues and cell lines. It's knockdown leads to inhibition of cell proliferation, mainly through inducing cellular apoptosis, both *in vivo* and *in vitro*. Importantly, we have shown that PAQR4 competes with Keap1 to interact with and to inhibit Nrf2 protein degradation. This activity is different from the adaptive role of PAQR3 facilitating Keap1-Nrf2 complex formation and protein degradation, and decreased expression of PAQR3 in NSCLC can also lead to chemoresistance by increasing Nrf2 stability 31. Consistent with this finding, we identified that PAQR3 is decreased in NSCLC. Furthermore, PAQR4 knockdown promotes the sensitivity of cancerous cell to DDP/ Paclitaxel treatment through decreasing Nrf2 protein levels.

The underlying mechanism of why PAQR4 behaves differently from PAQR3 in NSCLC is still unknown, and deciphering the expression regulation of these two family members under different cellular or tissue context might be able to resolve this mystery. For example, the promoter methylation levels or transcriptional regulation mechanisms may be different. Due to lack of commercial or homemade antibodies detecting PAQR4, we were unable to determine the protein expression patterns of PAQR4 both in cancerous tissues or cell lines. Therefore, whether endogenous PAQR4 interacts with Nrf2 and/or how PAQR4 is deregulated at post-translational level in NSCLC need further characterization. In addition, the physical interacting partners for PAQR3 and PAQR4 could also be different in a context dependent manner. Recent findings showed that PAQR4 promotes cell proliferation and metastasis through CDK4-pRB-E2F1 in NSCLC 33, although we also observed that PAQR4 regulates NSCLC cancerous cell proliferation, we concluded that PAQR4 knockdown inhibits cell proliferation by inducing cellular apoptosis instead of regulating CDK4 protein stabilities. Taken together, our findings identified PAQR4 as a potential therapeutic target for NSCLC treatment in the future.

## Material and Methods

### Plasmids construction and cell culture

Independent shRNAs targeting PAQR4 were constructed using a pLKO.1 vector. The two independent PAQR4 targeting sequences are: shRNA#1, 5'-GCAGGCTCCGTGCTCTATCAC-3'; shRNA#2, 5'-CGTCTTGCTCTGAGAGTTCAA-3'. The pNFE2L2 (NRF2)-ENTER (Gene ID: NM_006164) and pKEAP1-ENTER (Gene ID: NM_203500) plasmids were purchased from Vigene Biosciences Inc., and were sub-cloned into pCDNA3.1. Nrf2 was N-terminal tagged with 6×Myc, and Keap1 was N-terminal tagged with 3×HA. The full length cDNA of *PAQR4* (Gene ID: NM_152341.5) was synthesized by Shanghai Generay Biotech, and sub-cloned into pCDH-MSCV-E2F-eGFP lentiviral vector or pCDNA3.1 vector with a 3×Flag at the C-terminus. The lentiviruses were generated according to the manufacturer's protocol, stable cell lines were generated by lenti-viral infection. The BEAS-2B cell line was a gift from Dr. Hongbin Ji at SIBS, CAS, China. HEK-293T was obtained from ATCC. A549, H1299, H1975, H1650 were purchased from Cobioer, China with STR document, BEAS-2B, A549, H1650, H1975, H358, GLC-82 and SPC-A1 cells were cultured in RPMI1640 medium (Corning) supplemented with 10% fetal bovine serum (FBS) and 1% penicillin/streptomycin. H1299 and HEK-293T cells were cultured in DMEM medium (Corning).

### Immuno-precipitation, immunoblotting and quantitative real-time RT-PCR

To detect the association among PAQR4, Nrf2 and Keap1, indicated constructs were transfected into HEK-293T cells and the cell lysates were subjected to immunoprecipitation with Flag/Myc/HA antibodies. The precipitated proteins were detected with indicated antibodies by western blot. To detect the ubiquitin-conjugated exogenous Nrf2, the Myc-Nrf2, HA-Ub and PAQR4-Flag constructs were transfected into HEK-293T cells, and exposed to 20 μM MG132 for 9 h prior to lysis. Cell lysates were subjected to immuno-precipitation with Myc antibody and immunoblotting with HA antibody. Indicated cells treated with 100 μg/mL cycloheximide (CHX) were harvested at various time points, and the cell lysates were subjected to western blot. Indicated cells were lysed by RNAiso Plus (Takara Bio, Beijing, China, Cat# 108-95-2). Total RNA was extracted according to the manufacturer's protocol, and reverse transcribed using a PrimeScript RT reagent Kit (Takara Bio, Beijing, China, Cat# RR047A). Quantitative real-time PCR was performed by FastStart Universal SYBR Green Master Mix (Roche, Cat# 04194194001) using an Applied Biosystems 7500 machine. The primers and antibodies used in this study are shown in Table 5 and 6.

### Cell proliferation, BrdU incorporation, colony formation assays

Indicated cells were plated onto 12-well plates, the cell numbers were subsequently counted each day using an automatic cell analyzer countstar (Shanghai Ruiyu Biotech Co., China, IC 1000). Cells were cultured in 8-well plates for 24h, pulsed with 10μM BrdU (Abcam, Cat# ab142567) for 20 min, and fixed with 4% PFA (paraformaldehyde). Cells were then incubated with BrdU (Cell Signaling Technology, Cat# 5292s, dilution 1:1000) primary antibody followed by secondary antibody detection (Abclonal, Cat# 61303, dilution 1:500). Cell nuclei were stained with DAPI (4', 6-diamidino-2-phenylindole). For colony formation assay, indicated cells were seeded in agar medium in 6-well plate with 3×10^3^ cells per well supplemented with 2 mL 10% FBS cell culture medium, and the medium changed every 3 days for 2~3 weeks. Indicated cells were fixed with 4% PFA and stained with crystal violet.

### Xenograft tumor formation assay *in vivo*

Male nude mice aged 5 weeks were randomly divided into different groups, and were then injected with 2 independent PAQR4 knockdown cell lines or scramble control shRNA cells, (1×10^6^ cells/ subcutaneously). Nude mice were monitored every day, xenograft tumor weights and volumes were measured with a sliding caliper every two days, and tumor volumes were calculated using the formula (L×W^2^)/2. All mice were sacrificed at the end of the experiment and the tumors were harvested and weighted. For the xenograft tumor cisplatin sensitivity assay, when the xenograft tumors reached to 50 mm^3^ of volume, the mice were injected with 7 mg/kg DDP by intraperitoneal injection once per week. Four weeks later, all mice were sacrificed at the end of the experiment and the tumors were harvested and weighted.

### SRB and drug sensitivity assays

Cells were plated into 96-well plates with 1×10^4^ cells per well and cultured overnight, indicated drugs were added. Cell viabilities were detected by sulforhodamine B (SRB) staining following a standard protocol. The assay was repeated in triplicate. Cells were plated into 6 cm culture dishes with 5×10^5^ cells per dish and cultured overnight, indicated drugs were added for 48 h. The apoptotic cell numbers were analyzed by flow cytometry. Indicated cell lysates were detect by antibodies against apoptotic markers by western blot.

### Immunofluorescence and Immunohistochemistry staining

To detect the co-localization of PAQR4 with Nrf2 or Keap1, indicated constructs were transfected into SPC-A1 cells, and the cells were seeded into 8-well plates with 5×10^4^ cells per well. 48 h after transfection, indicated cells were fixed with 4% paraformaldehyde for 20 min at room temperature, permeated with 0.2% Triton X-100 for 5 min, followed by primary and secondary antibody staining. Cells were again washed, mounted, air-dried, and then visualized by Nikon A1 confocal microscopy. For IHC staining, tumor sections were incubated with 3% hydrogen peroxide, blocked with 10% goat serum in 0.2% Triton X-100 containing PBS (phosphate buffer saline) and followed by primary antibody incubation. The biotin conjugated secondary antibody detection was performed at room temperature for 40 min, followed by diaminobenzidine (DAB) staining. Images were captured using a binocular Nikon research light microscope (ECLIPSE, Nikon) in bright field. The percentage of positive cells was counted using Image pro plus 6.0, and the percentage of positive cells in each image was calculated using the following formula: number of positive cells/number of all cells.

## Supplementary Material

Supplementary figures.

## Figures and Tables

**Table 1:** The abbreviation of cancer types used in supplementary Figure 1A.

Abbreviation	Full name of cancer
BLCA	Bladder urothelial carcinoma
BRCA	Breast invasive carcinoma
COAD	Colon adenocarcinoma
HNSC	Head and Neck squamous cell carcinoma
KICH	Kidney Chromophobe
KIRC	Kidney renal clear cell carcinoma
KIRP	kidney renal papillary cell carcinoma
LIHC	Liver hepatocellular carcinoma
LUAD	Lung adenocarcinoma
LUSC	Lung squamous cell carcinoma
PRAD	Prostate adenocarcinoma
THCA	Thyroid carcinoma
UCEC	Uterine Corpus Endometrial Carcinoma

**Table 2:** Clinicopathological characteristics of patients with non-small cell lung cancer (NSCLC) and noncancerous lung tissues in the tissue arrays

SCC (n=129)			ADC (n=129)	
Characteristics	No. of patients (%)		Characteristics	No. of patients (%)
**Age(years)**			Age(years)	
≤50	31(24.0)		≤50	36(27.9)
>50	98(76.0)		>50	93(72.1)
**Gender**			Gender	
Male	118(91.5)		Male	76(58.9)
Female	11(8.5)		Female	53(41.1)
**Clinical stages**			Clinical stages	
Stage I	29(22.5)		Stage I	40(31.0)
Stage II	29(22.5)		Stage II	27(20.9)
Stage III	63(48.8)		Stage III	54(41.9)
Stage IV	8(6.2)		Stage IV	8(6.2)
**LNM status**			LNM status	
N0	57(44.2)		N0	53(41.1)
N1/N2/N3	72(55.8)		N1/N2/N3	76(58.9)
**Differentiation**			Differentiation	
Well	3(2.3)		Well	2(1.6)
Moderate	56(43.4)		Moderate	57(44.2)
Poor	70(54.3)		Poor	70(54.3)
**Survival status**			**Survival status**	
Live	82(63.6)		Live	74(57.4)
Death	47(36.4)		Death	55(42.6)
			
**Non-cancerous lung tissues (n=49)**				
**Age (years)**			**Gender**	
≤50	39(79.6)		Male	25(51.0)
>50	10(20.4)		Female	24(49.0)

**Table 3:** PAQR4 mutation information in human NSCLC cancers

Sample ID	Cancer Study	AA change	Type
LUAD-S01315	Lung Adenocarcinoma (Broad, Cell 2012)	W72C	Missense
LUAD-S01315- Tumor	Pan-Lung Cancer (TCGA, Nat Genet 2016)	W72C	Missense
TCGA-63-6202-01	Pan-Lung Cancer (TCGA, Nat Genet 2016)	G178E	Missense
TCGA-63-6202-01	Lung Squamous Cell Carcinoma (TCGA, Provisional)	G178E	Missense
TCGA-63-6202-01	Lung Squamous Cell Carcinoma (TCGA, Nature 2012)	G178E	Missense
TCGA-63-6202-01	Lung Squamous Cell Carcinoma (TCGA, PanCancer Atlas)	G178E	Missense
TCGA-56-7579-01	Pan-Lung Cancer (TCGA, Nat Genet 2016)	L165Cfs*71	Frame_Shift _Ins
TCGA-56-7579-01	Lung Squamous Cell Carcinoma (TCGA, PanCancer Atlas)	L165Cfs*71	Frame_Shift _Ins
LUAD-E00897	Lung Adenocarcinoma (Broad, Cell 2012)	Q241*	Nonsense
TCGA-37-3789-01	Lung Squamous Cell Carcinoma (TCGA, Nature 2012)	R184S	Missense
CRUK0001-R1	Non-Small Cell Lung Cancer (TRACERx, NEJM 2017)	P200A	Missense
CRUK0001-R1	Non-Small Cell Lung Cancer (TRACERx, NEJM 2017)	R184C	Missense
CRUK0001-R3	Non-Small Cell Lung Cancer (TRACERx, NEJM 2017)	R184C	Missense

**Table 4:** The cBioPartal database query used in Figure 1D.

Cancer type	Cancer study	DOI	Journal
LUAD(#1)	Lung Adenocarcinoma (Broad, Cell 2012)	doi: 10.1016/j.cell.2012.08.029	Cell
SCLC	Small cell lung cancer(Johns Hopkins, Nat Genet 2012	doi: 10.1038/ng.2405	Nat Genet*
LUSC(#1)	Lung Squamous Cell Carcinoma (TCGA, Nature 2012)	doi: 10.1038/nature11404	Nature
LUSC(#2)	Lung Squamous Cell Carcinoma (TCGA, Provisional)	TCGA	
NSCLC	Pan-lung cancer(TCGA, Nat Genet 2016)	doi: 10.1038/ng.3564	Nat Genet*
LUSC(#3)	Lung Squamous Cell Carcinoma (TCGA, PanCancer Atlas)	doi: 10.1016/j.cell.2018.02.052	Cell
LUAD(#2)	Lung Adenocarcinoma (TCGA, Provisional)	TCGA	
Tracerx	Non-Small Cell Lung Cancer (TRACERx, NEJM 2017)	doi: 10.1056/NEJMoa1616288	NEJM^#^
LUAD(#3)	Lung Adenocarcinoma (TCGA, Pancan)	doi: 10.1016/j.cell.2018.02.052	Cell
LUAD(#4)	Lung Adenocarcinoma (TCGA, Nature 2014)	doi: 10.1038/nature13385	Nature

*Nat Genet=Nature Genetics; ^#^NEJM= The New England journal of medicine

**Table 5:** Primer sequences used in this study.

Name	Primer (5'-3')
human PAQR4_qPCR_F	CGAACTGGGCAACATCTACA
human PAQR4_qPCR_R	AGGGTGTTGACAAGGCAGAC
human Actin_qPCR_F	AAGTGTGACGTGGACATCCGC
human Actin_qPCR_R	CCGGACTCGTCATACTCCTGCT
human Nrf2_qPCR_F	TGCCCCTGGAAGTGTCAAACA
human Nrf2_qPCR_R	CAACAGGGAGGTTAATGATTT
human GCLC_qPCR_F	ATGCCATGGGATTTGGAAT
human GCLC_qPCR_R	AGATATACTGCAGGCTTGGAATG
human GCLM_qPCR_F	GACAAAACACAGTTGGAACAGC
human GCLM_qPCR_R	CAGTCAAATCTGGTGGCATC
human GR_qPCR_F	CACGGAGGAGCTGGAGAAC
human GR_qPCR_R	CGACAAAGTCTTTTTAACCTCCTT
human TR_qPCR_F	CAGACGGGGAGGCTTTTC
human TR_qPCR_R	CCGAGAGCGTTCCTTTCA
human NQO1_qPCR_F	ATGTATGACAAAGGACCCTTCC
human NQO1_qPCR_R	TCCCTTGCAGAGAGTACATGG
human PAQR3_qPCR_F	AACCCGTACATCACCGACG
human PAQR3_qPCR_R	TCTGGACGCACTTGCTGAAG

**Table 6:** Antibodies used in this study.

Name	Catalog number	Dilution	Supplier	species
CDK2	10122-1-AP	1:1000	Proteintech	Rabbit
CDK4	ab108357	1:1000	abcam	Rabbit
LaminB	ab16048	1:2000	abcam	Rabbit
β-actin	60008-1-1g	1:5000	Proteintech	Mouse
GAPDH	60004-1-Ig	1:10000	Proteintech	Mouse
PARP	9542S	1:500	CST	Rabbit
Cleaved Caspase3	9661S	1:500	CST	Rabbit
Bcl-2	15071S	1:1000	CST	Mouse
Bax	ab77566	1:1000	abcam	Mouse
Brdu	5292	1:1000	CST	Mouse
Nrf2	ab62352	1:1000	abcam	Rabbit
Flag	14793	1:1000	CST	Rabbit
Flag	1804	1:1000	sigma	Mouse
Myc	sc-40	1:1000	santa cruz	Mouse
Myc	sc-789	1:1000	santa cruz	Rabbit
HA	sc-7392	1:1000	santa cruz	Mouse
PAQR4	13401-1-AP	1:100	Proteintech	Rabbit
