# Supplementary material for: PAQR4 promotes chemoresistance in non-small cell lung cancer through inhibiting Nrf2 protein degradation
Source: Theranostics. 2020 Feb 19;10(8):3767–78. doi: 10.7150/thno.43142 (PMC7069097; doi:10.7150/thno.43142)
Supplement: Supplementary file 1 — Supplementary figures. [file thnov10p3767s1.pdf]

Supplementary figures and figure legends

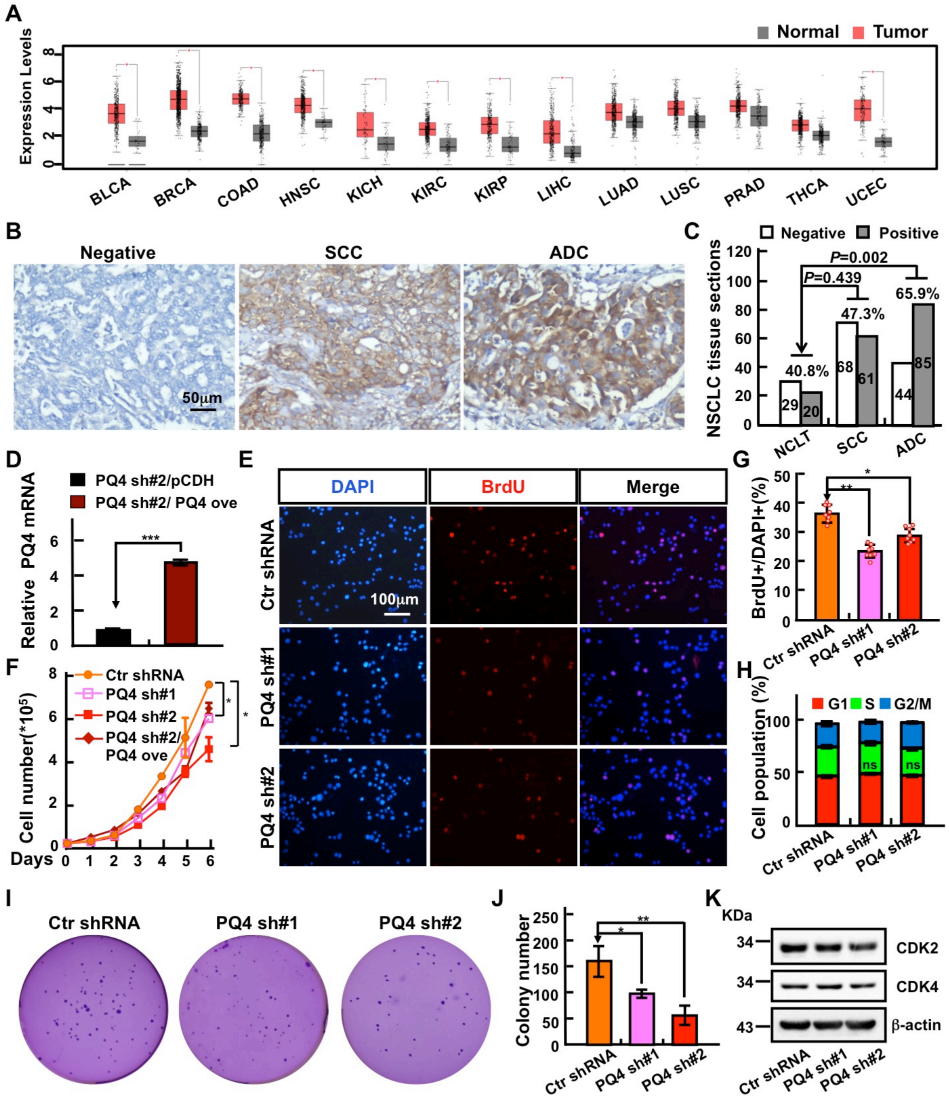

Figure S1. PAQR4 regulates NSCLC cancerous cell proliferation. (A) PAQR4 is highly expressed in various cancer types, based on the RNA-seq data from TCGA. The boxes show the median and interquartile range, and the whiskers show the minimum and maximum. **(B-C)** PAQR4 protein expression pattern was examined in lung squamous cell carcinoma (SCC) or lung adenocarcinoma (ADC) tissue using IHC staining. The negative control was control IgG staining in the lung adenocarcinoma tissue. (C) Quantification data for (B). Scale bar: 50 μm. **(D)** Validation of PAQR4 overexpression in GLC-82 by real-time RT-PCR. **(E)** PAQR4 Knockdown dramatically inhibits GLC-82 cell proliferation, which can be reversed by PAQR4 overexpression**. (F-G)** Knockdown of PAQR4 decreases DNA synthesis in GLC-82 cells as determined by BrdU incorporation assay. (G) is the quantification data for (F). Scale bar: 100

μm. **(H)** PAQR4 knockdown in GLC-82 has no effect on cell cycle as measured by flow cytometry. Quantification data is shown. **(I-J)** Knockdown of PAQR4 decreases colony formation ability of GLC-82 cells. (J) is the quantification data for (I). **(K)** PAQR4 does not regulate protein expressions of CDK2 or CDK4 in GLC-82 cells. Means±SEM, * P <0.05; ** P <0.01; *** P <0.001; P=ns: no significant difference; t-test.

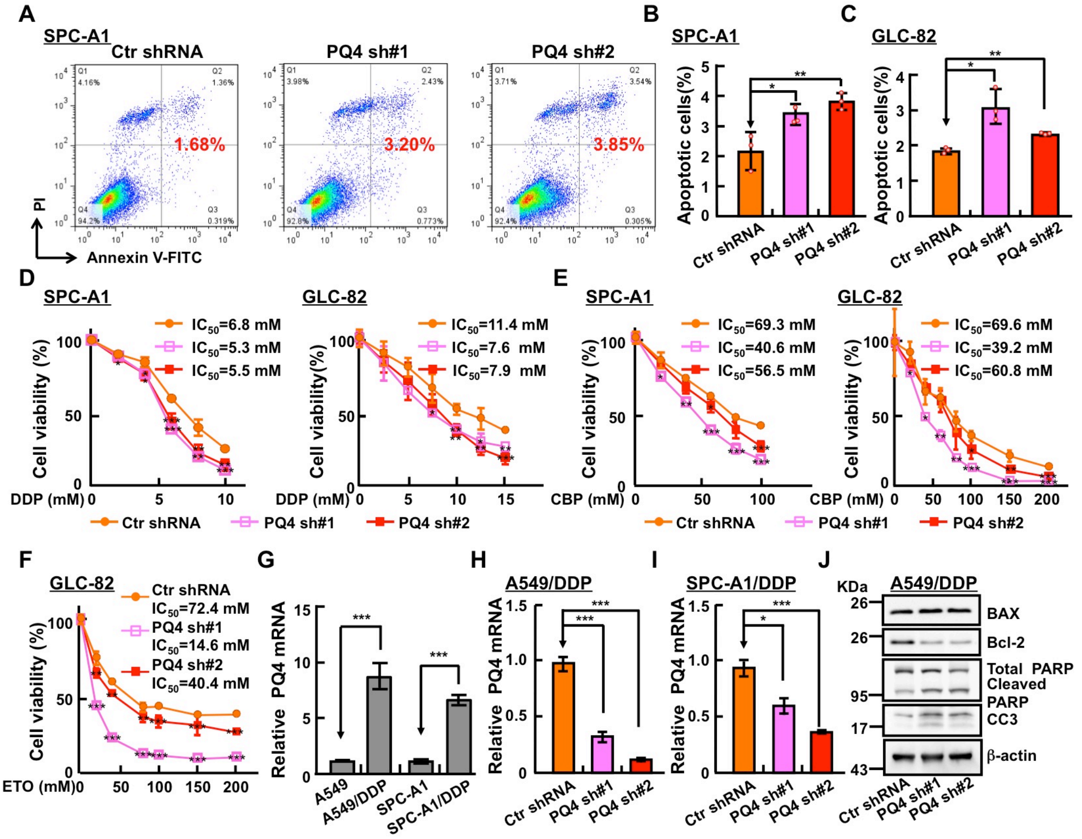

Figure S2. PAQR4 knockdown promotes cellular apoptosis. (A-C) PAQR4 knockdown promotes cellular apoptosis in both SPC-A1 (A-B) and GLC-82 (C). **(D-E)** Effect of DDP (D) and carboplatin (CBP: E) on cell viability of indicated SPC-A1 and GLC-82 cell lines by SRB assay. Individual IC_{50} is indicated. **(F)** Etoposide (ETO) induced cellular apoptosis was also tested in PAQR4 knockdown GLC-82 cells. Individual IC_{50} is indicated. **(G)** PAQR4 is highly expressed in A549/DDP cells and SPC-A1/DDP cells compared to A549 and SPC-A1, by real-time RT-PCR. **(H-I)** Establishment of PAQR4 knockdown in A549/DDP and SPC-A1/DDP cells. **(J)** A549/DDP cells were treated with DDP (20 μM) for 48 h, and indicated total extracts were probed with indicated antibodies: PARP, CC3, Bcl-2, BAX and β-actin. Means±SEM, * P <0.05; ** P <0.01; *** P <0.001; t-test.

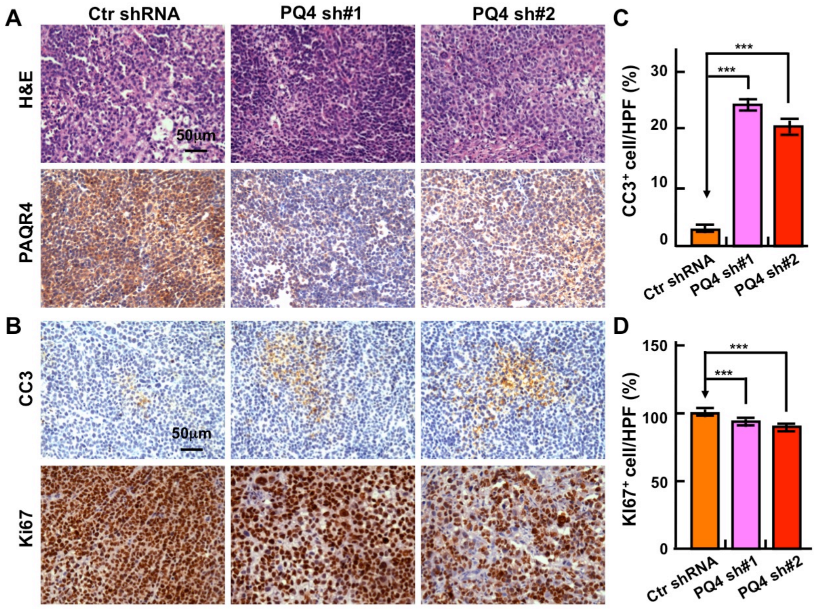

Figure S3. PAQR4 knockdown inhibits xenograft tumor formation in vivo. (A) Representative images of H&E-stained and IHC staining of xenograft tumor sections. Scale bar: 50 μm. **(B-D)** Representative IHC staining of Cleaved Caspase 3 (CC3) and Ki67 for indicated xenograft tumors without DDP treatment (B). The quantification data of CC3 (C) and Ki67 (D) are included. Scale bar: 50 μm. Means±SEM, * $P < 0.05$; ** $P < 0.01$; *** $P < 0.001$; *t*-test.

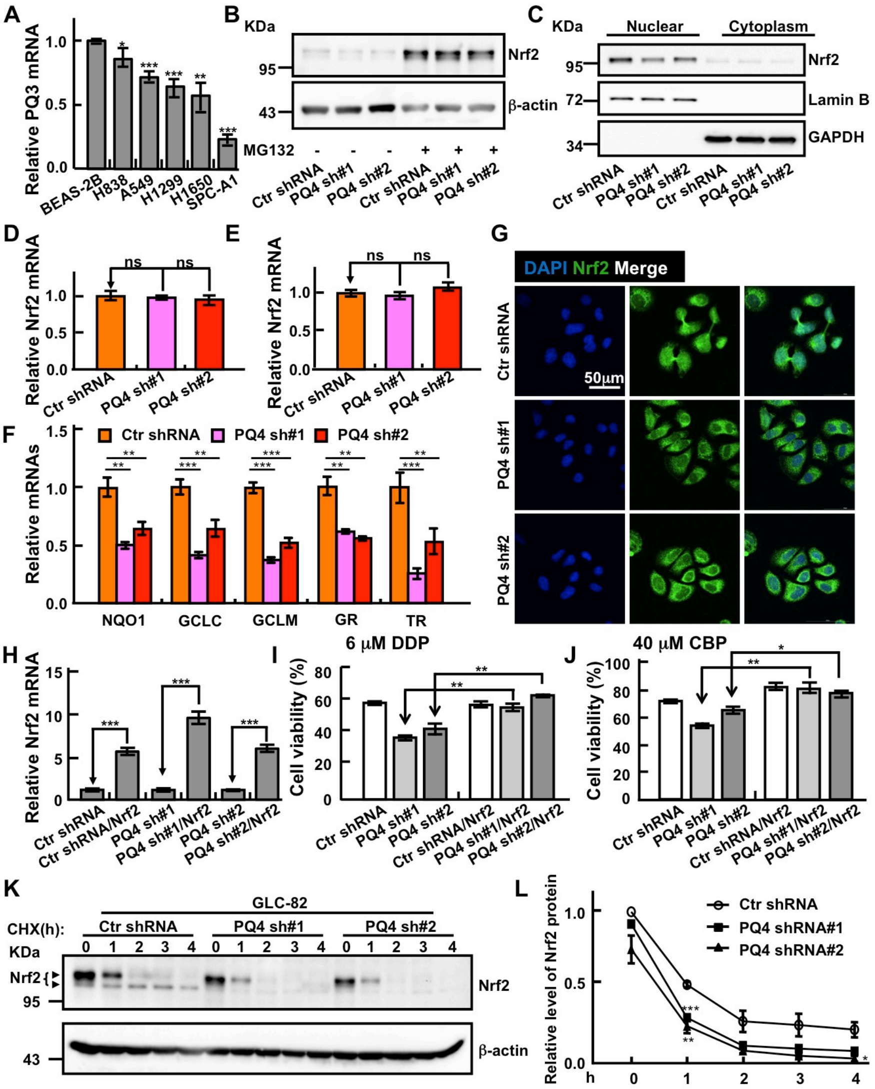

Figure S4. PAQR4 promotes Nrf2 nuclear translocation. (A) PAQR3 is downregulated in NSCLC cancerous cell lines compared to control BEAS-2B by real-time RT-PCR analysis. PQ3=PAQR3. **(B-C)** Indicated extracts were probed with indicated antibodies by western blot: Nrf2, β-actin, Lamin B (nuclear fraction), GAPDH (cytosol fraction). **(D-F)** Relative mRNA expression of Nrf2 in SPC-A1 (D) and GLC-82 cell lines (E) and its downstream genes (F) after PAQR4 knockdown by real-time RT-PCR analysis in GLC-82 cells. **(G)** Representative images of Immunofluorescent staining of Nrf2 in GLC-82 cells. **(H-J)** PAQR4 knockdown promotes DDP or CBP induced cellular apoptosis in SPC-A1 cells, which can be reversed by Nrf2 overexpression. (H) Validation of Nrf2 overexpression in indicated cells. Indicated cell viabilities were examined by SRB assay upon DDP (I) or CBP (J) treatment. **(K-L)** Indicated cells were treated by cycloheximide (CHX: 100 µg/mL) and indicated cell lysates were examined by western blot with indicated antibodies. (L) is the quantification data for (K).

Means±SEM, * *P* <0.05; ** *P* <0.01; *** *P* <0.001; *P*=ns: no significant difference; *t*-test.

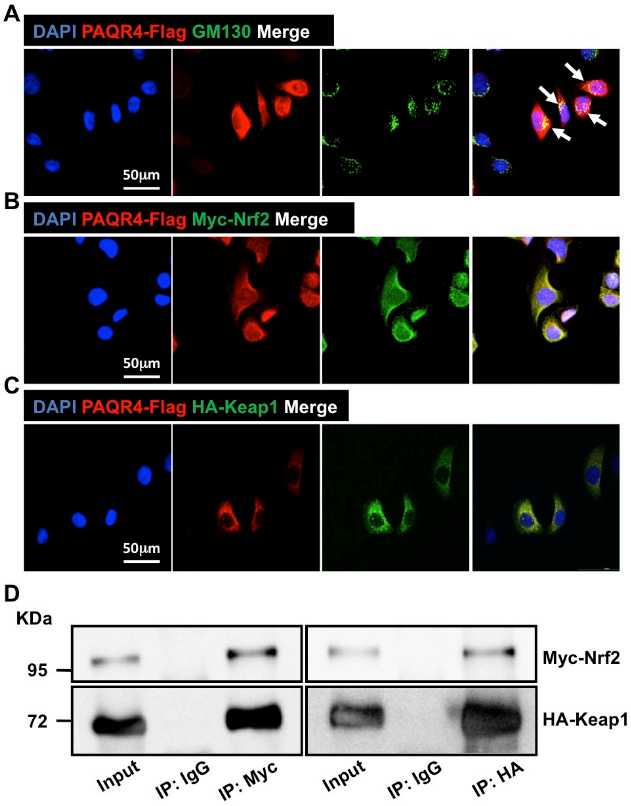

Figure S5. Subcellular localization of PAQR4, Nrf2 and Keap1. (A-C) SPC-A1 cells were transiently transfected with indicated plasmids: Flag-tagged PAQR4 (A) with Myc-tagged Nrf2 (B) or HA-tagged Keap1 (C). 48 h after transfection. The cells were examined by immunofluorescent staining and confocal microscopy. The Golgi apparatus was stained with GM130, and the nuclei were stained with DAPI. The arrows indicate apparent localization of PAQR4 to the Golgi apparatus. Scale bar: 50 μm. **(D)** Interaction of Nrf2 and Keap1. HEK-293T cells were transfected with indicated plasmids and the cell lysates were used for IP and IB.
